# Experimental study on the effects of ghrelin on gastric smooth muscle and posterior limb skeletal muscle in mice

**DOI:** 10.3389/fmed.2025.1631707

**Published:** 2025-07-28

**Authors:** Ping Wang, Cunbo Yu, Yibing Li, Xiaoxiao Zhang, Xueli Yao, Yingjian Zhang

**Affiliations:** ^1^College of Basic Medicine and Forensic Medicine, Henan University of Science and Technology, Luoyang, Henan, China; ^2^Department of Gastroenterology, The First Affiliated Hospital, College of Clinical Medicine, Henan University of Science and Technology, Luoyang, Henan, China; ^3^Henan Medical Key Laboratory of Gastrointestinal Microecology and Hepatology, Luoyang, Henan, China

**Keywords:** ghrelin, disuse muscle atrophy, PI3K-Akt-mTORC1, gastric smooth muscle, constipation

## Abstract

**Introduction:**

This study aims to systematically explore the regulatory effects of ghrelin on hindlimb skeletal muscle function and gastrointestinal smooth muscle in mice. The objective is to elucidate the improvement effects of ghrelin on functional constipation through regulating skeletal muscle function and gastrointestinal motility, providing new theoretical support for the prevention and treatment of functional constipation.

**Methods:**

Dexamethasone-induced *in vitro* cell models and tail suspension-induced *in vivo* mouse models were employed to analyze the regulatory effect of ghrelin on the PI3K-Akt-mTORC1 signaling pathway. Additionally, a loperamide-induced constipation mouse model was used to evaluate the effects of ghrelin on fecal output, gastric motility, and smooth muscle activity. Experimental techniques included Western blotting, enzyme-linked immunosorbent assay (ELISA), histopathological staining, phenol red assay, and quantitative analyses of Ca^2+^ and ATP to comprehensively assess the impact of ghrelin on muscle atrophy and gastrointestinal function.

**Results:**

The results showed that, *in vitro*, ghrelin significantly upregulated the expression of p-AKT and reduced the levels of p-Foxo3a, effectively alleviating muscle atrophy. *In vivo*, the muscle condition of mice was improved and the expression of atrophy-related proteins (MAFbx and MuRF1) decreased, promoting the functional recovery of hindlimb muscles. In constipated mice, ghrelin increased fecal water content and defecation frequency, and accelerated gastric emptying, findings consistent with elevated ghrelin levels in serum and tissue. Moreover, ghrelin promoted calcium ion influx and ATP production in gastric smooth muscle cells, thereby enhancing gastrointestinal motility.

**Discussion:**

In conclusion, ghrelin effectively alleviates muscle atrophy by activating the PI3K-Akt-mTORC1 signaling pathway, and improves gastrointestinal motility by enhancing smooth muscle activity. These findings highlight ghrelin’s potential as an effective therapy for functional constipation.

## 1 Background

Ghrelin was initially identified as a polypeptide consisting of 28 amino acid residues and was isolated from endocrine cells in the stomach. It is renowned for its role in stimulating food intake by acting on the hypothalamus (1). Studies have demonstrated that ghrelin, as an endogenous hormone, not only enhances appetite but also regulates gastrointestinal motility, blood glucose levels, memory and cognitive functions, sleep–wake cycles, and mood, among others (2–6). Ghrelin is also capable of promoting the secretion of growth hormone and regulating feeding behavior, energy balance, and gastrointestinal peristalsis (7–9). However, the alleviating effect of ghrelin on disuse muscle atrophy and its mechanism remain unclear.

Functional constipation is a common gastrointestinal disorder, whose clinical manifestations include difficult defecation, reduced frequency of defecation, and hard stools. Abnormal movement of the skeletal muscle groups in the pelvic region may lead to a decrease in the strength and coordination of the muscles in this area, thereby exacerbating the symptoms of constipation. This physiological change is particularly common among the elderly and patients who have been bedridden for a long time, significantly affecting their quality of life (10). Gastrointestinal smooth muscles promote the movement of food and feces by maintaining normal peristaltic function and are regulated by the autonomic nervous system (including the sympathetic and parasympathetic nerves) to ensure the coordination and effective execution of peristaltic movements. However, when the peristaltic function of smooth muscles weakens or neural signal conduction is abnormal, feces may stay in the intestinal tract for an overly long time, resulting in excessive water absorption, hardening of feces, further aggravating defecation difficulties, and ultimately causing constipation. Modern research indicates that skeletal muscle loss and gastrointestinal motility disorders play important roles in the onset and progression of functional constipation (11). They jointly exacerbate the symptoms of constipation and affect the quality of life of patients by influencing key steps in the defecation process, including muscle coordination and gastrointestinal peristalsis. Currently, the treatment of functional constipation usually involves drug intervention, aiming to improve intestinal peristalsis and increase the frequency of defecation. Nevertheless, the therapeutic effects of the available drugs are limited, and they may be accompanied by side effects, making it impossible for some patients to obtain satisfactory symptom relief. Additionally, many drugs only target the symptoms rather than the fundamental mechanisms of the disease, thus their long-term efficacy and improvement in the quality of life are often insufficient. Modern research still faces challenges in exploring the pathophysiological mechanisms of this disease, especially in the pathways related to muscle dysfunction. Therefore, finding methods to improve disuse muscle atrophy and gastrointestinal motility disorders holds significant clinical significance.

Therefore, in this study, by constructing cell and mouse models of disuse muscle atrophy and constipation, we aim to verify the improvement of gastrointestinal function in disuse muscle atrophy and constipated mice through the secretion of ghrelin. We will investigate the role of ghrelin in regulating the PI3K-Akt-mTORC1 signaling pathway in skeletal muscle in disuse muscle atrophy in mice and its effect on improving constipation in mice by increasing the expression of Ca2 + channels and ultimately enhancing the contraction of smooth muscles. Through this research, it is expected that ghrelin or its derivatives can become new drug candidates for the treatment of functional constipation in the future, helping patients restore normal intestinal motility and improve their quality of life.

## 2 Materials and methods

### 2.1 Materials and reagents

The mouse ghrelin enzyme-linked immunosorbent assay (ELISA) kit was acquired from Enzyme-linked Biotechnology Co.; the ATP enzyme kit was sourced from Jiangsu Kaiji Biological Technology Development Co., Ltd.; C57BL/6 mice were obtained from Shanghai Slack Laboratory Animal Co., Ltd.; ICR mice were procured from Suzhou Sibeifu Biotechnology Co., Ltd.; the laser confocal dish purchased from Hangzhou Shunyoo Biotechnology Co., Ltd.; the Fluo-3 AM detection kit was sourced from Jiangsu Kaiji Biotechnology Co., Ltd.; C2C12 cells, Western Blotting detection kit, and Hematoxylin–Eosin staining kit were acquired from Jiangsu Kaiji Biotechnology Research Institute. Antibodies against MAFbx, MuRF1, Akt, p-Akt, FoxO3a, and p-FoxO3a were obtained from Annoron Biotechnology Co., Ltd.; rapamycin and ouabain were procured from Shanghai Aladdin Biochemical Technology Co., Ltd.

### 2.2 Cell experiments and grouping

C2C12 myoblasts were cultivated in DMEM high-glucose medium containing 10% fetal bovine serum, 2 mmol/L glutamine, 50 μg/mL penicillin and streptomycin, and placed in an incubator at 37°C and 5% CO_2_ until the myoblasts reached the logarithmic phase. Mononuclear myoblasts were differentiated and fused into tubular multinucleated myotubes in differentiation medium containing 2% horse serum, 2 mmol/L glutamine, 50 μg/mL penicillin and streptomycin. An *in vitro* disuse muscle atrophy model was induced by the synthetic glucocorticoid dexamethasone (1 μM, 24 h).

The experimental groups were as follows: C2C12 myotube blank group, model group, model + 10 nM ghrelin, model + IGF-1 (10 ng/mL), model + ghrelin 10 nM + rapamycin (1 μg/mL), model + ghrelin 10 nM + rapamycin (10 μg/mL), model + ghrelin 10 nM + rapamycin (100 μg/mL), model + ghrelin 10 nM + rapamycin (1 mg/mL), model + ghrelin 10 nM + wortmannin (1 μg/mL), model + ghrelin 10 nM + wortmannin (10 μg/mL), model + ghrelin 10 nM + wortmannin (100 μg/mL), model + ghrelin 10 nM + wortmannin (1 mg/mL), among which rapamycin and wortmannin were pathway inhibitors. The modeling lasted for 24 h, and the drug treatment lasted for 24 h.

### 2.3 Animal experiment groups and model establishment

#### 2.3.1 Mouse model of disuse muscle atrophy

**Figure 1:**
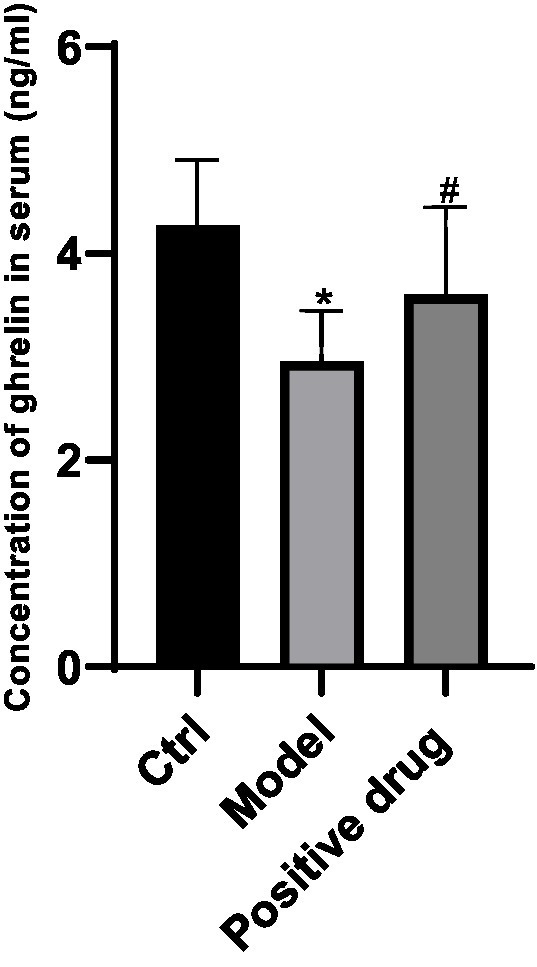
Ghrelin concentration in the blank control group, model group, and positive drug group. ^*^p < 0.05, compared with the blank control group; ^#^*p* < 0.05, compared with the Positive drug group.

**Figure 2:**
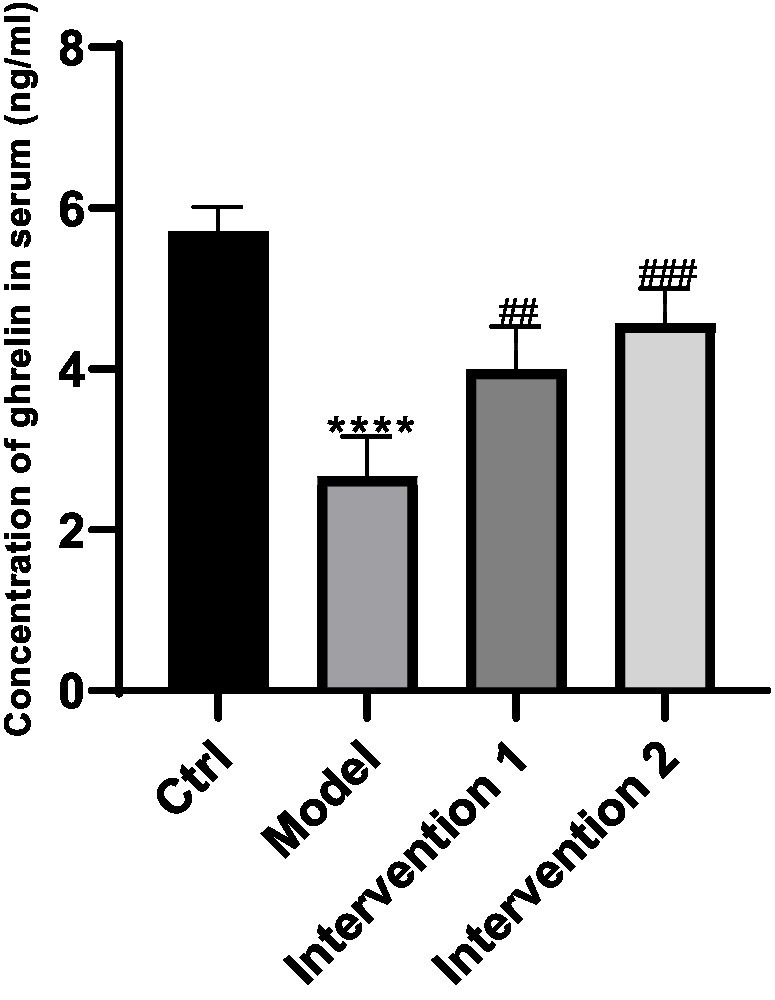
Changes in ghrelin secretion in serum. ^****^P < 0.0001, compared with the blank control group; ^##^*P* < 0.01, ^###^*P* < 0.01, compared with the model group.

**Figure 3:**
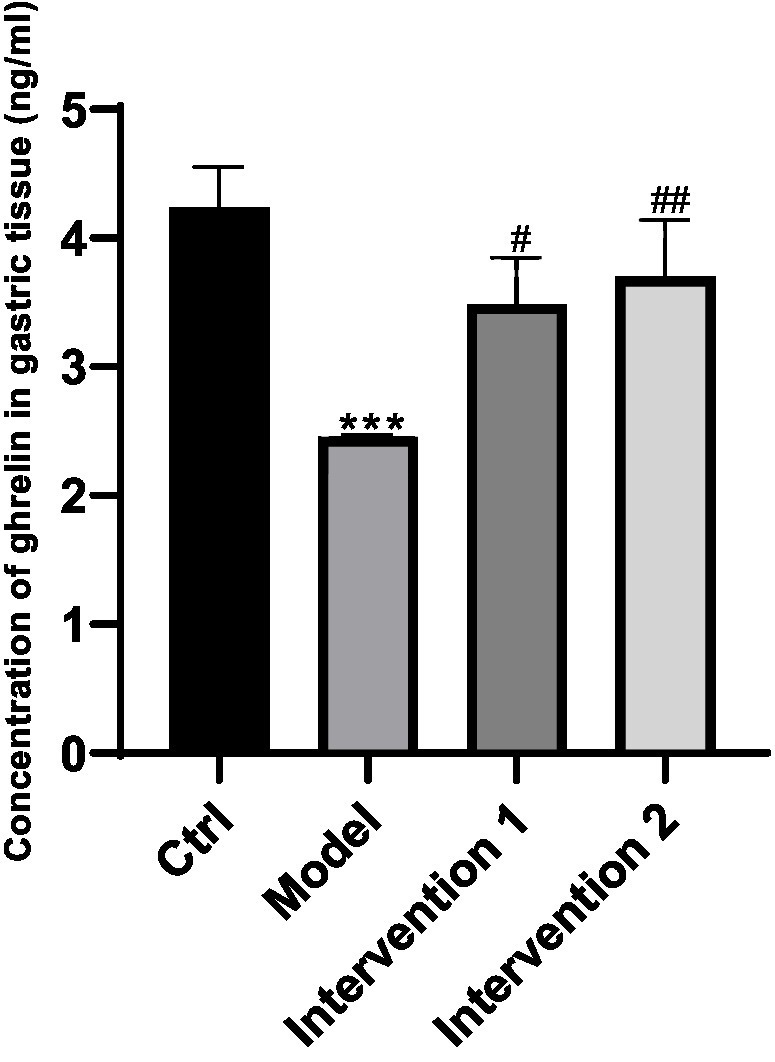
Changes of ghrelin secretion in gastric tissues. ^***^P < 0.001, compared with the blank control group; ^#^*P* < 0.05, ^##^*P* < 0.001, compared with the model group.

Eighteen male C57BL/6 mice with a body weight between 18 and 20 g were selected. The housing room temperature was controlled at 24–26°C, the humidity was 10–30%, and the mice had free access to drinking water and food. After 1 week of adaptive feeding, they were randomly stratified by body weight and divided into three groups. They were randomly assigned to the blank group (6 mice), the model group (6 mice), and the positive drug group (Shouhui Tongbian Capsules at 90 mg/kg) (6 mice). Shouhui Tongbian Capsules, a traditional Chinese patent medicine for constipation treatment, can promote intestinal motility by regulating the intestinal flora. The results of both the main experiment and the preliminary studies showed that administration of Shouhui Tongbian Capsules could increase ghrelin levels; therefore, it was used as an intervention drug. The relevant results are shown in Figures 1–3.

**Figure 4:**
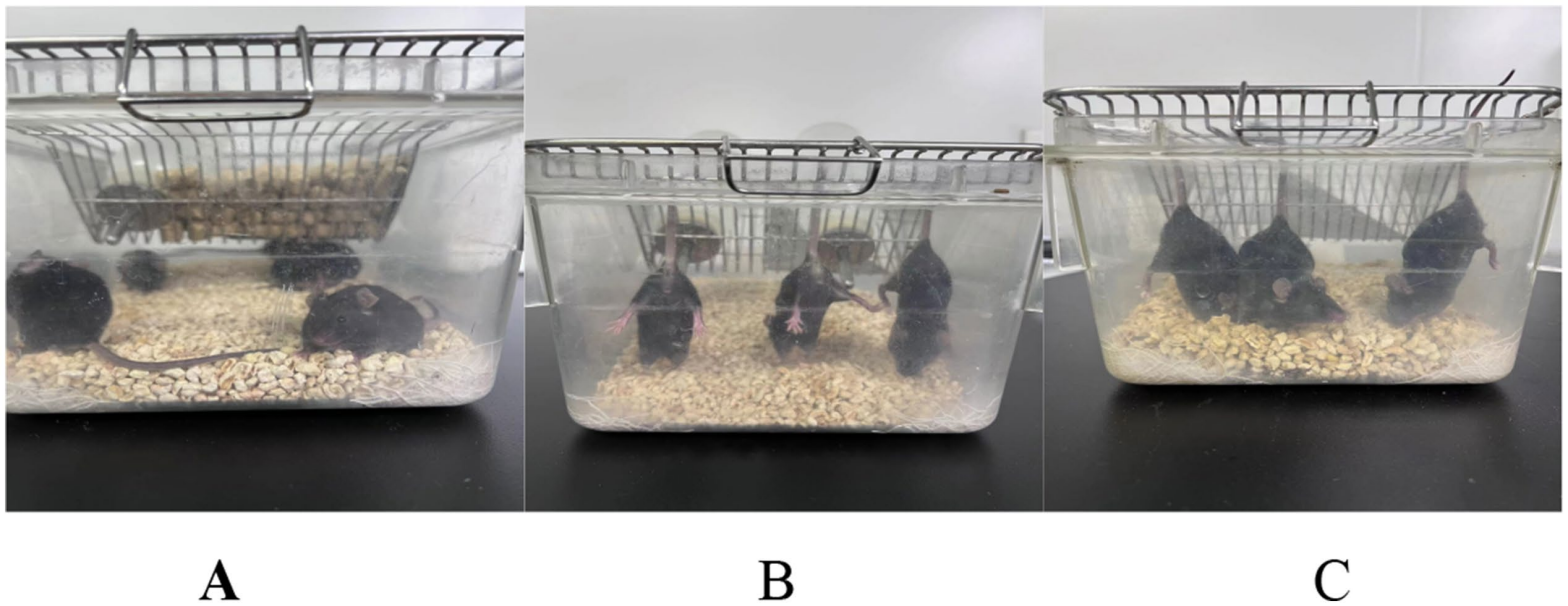
Tail suspension of mice in each. **(A)** Blank; **(B)** Model; **(C)** Positive drug.

In this experiment, a mouse model of disuse muscle atrophy was established using the tail suspension method. This method is widely used to simulate unloading/disuse conditions and to induce disuse muscle atrophy, reliably causing physiological and behavioral changes associated with specific diseases or conditions. The procedure was performed as described in references (10, 11). Except for the blank control group, all other groups of mice were subjected to tail suspension in specialized cages. Specifically, the distal end of the mouse tail was fixed to the top of the cage with adhesive tape, ensuring that the forelimbs could touch the cage floor while the hindlimbs remained suspended. The body axis of the mouse formed an angle of approximately 30° with the horizontal plane, and the mice were allowed to rotate freely 360°. Throughout the modeling period, the mice had free access to food and water to maintain basic physiological needs. Tail suspension was maintained for consecutive 4 weeks. The results of the tail suspension model are shown in Figure 4. After modeling, mice in the drug intervention group were administered Shouhui Tongbian Capsules by gavage at a dose of 60 mg/kg once daily for consecutive 7 days. Mice in the model group received an equivalent volume of physiological saline by gavage once daily for consecutive 7 days.

#### 2.3.2 Mouse model of constipation

In this experiment, 24 healthy male ICR mice aged 8 weeks (body weight 19 ± 0.6 g) were used. The mice were housed under a 12-h light/12-h dark cycle at a temperature of 24–26°C and a relative humidity of 10–30%. After 1 week of acclimatization, the mice were randomly divided into four groups, with six mice in each group: normal control group, model group, intervention group 1, and intervention group 2. According to methods described in references (12, 13), mice in the model group and both intervention groups were administered loperamide orally by gavage at a dose of 10 mg/kg (split into two doses, morning and evening) for 14 consecutive days to establish a slow transit constipation (STC) mouse model. Loperamide is an opioid receptor agonist that can inhibit intestinal peristalsis, delay gastrointestinal transit, and promote water absorption in the colon; thus, it is widely used in rodent constipation models (12). Mice in the normal control group received an equal volume of normal saline by gavage daily for 21 consecutive days. Mice in the model group were given loperamide for 14 days to induce constipation, followed by normal saline for another 7 days by gavage. After 14 days of loperamide administration, mice in intervention group 1 were treated with domperidone at a dose of 0.42 mg/kg once daily by gavage for 7 consecutive days. Mice in intervention group 2, after the loperamide administration, were given Shouhui Tongbian Capsules at a dose of 60 mg/kg by gavage once daily for 7 consecutive days.

### 2.4 Comprehensive assessment of general indicators in mice

#### 2.4.1 Changes in body weight and wet weight of hind limb muscles in mice with disuse muscle atrophy

The levels of ghrelin secretion in the serum of C57BL/6 and ICR mice, as well as in the gastric tissue of ICR mice, were assessed using the enzyme-linked immunosorbent assay (ELISA). All samples were collected after a 24-h fasting period following the final administration, to minimize the influence of food intake on ghrelin levels. Initially, serum samples from C57BL/6 and ICR mice, along with gastric tissue samples from ICR mice, were collected and processed in strict accordance with the kit instructions. The samples were incubated with enzyme conjugates, and after a series of washing steps, a substrate solution was added to initiate color development. Absorbance was then measured at a specific wavelength with a microplate reader to determine the concentration of ghrelin. To ensure the reliability and accuracy of the results, each sample group was tested in triplicate.

During the experimental modeling phase, the body mass of each group of C57BL/6 mice was measured every 3 days. Upon completion, the soleus muscle was isolated, washed with physiological saline to remove excess fat and non-muscle tissues, and thoroughly blotted dry with absorbent paper. The wet weight of the muscle was subsequently recorded using an electronic balance.

#### 2.4.2 Variations in physiological indicators and food intake in constipated mice

Changes in body weight, frequency of defecation, number of fecal pellets, and moisture content were documented for each group of ICR mice. Food intake variation was monitored at specific intervals (0–1 h, 1–2 h, 2–4 h, 4–24 h) over a 24-h period.

#### 2.4.3 Assessment of gastric emptying rate in constipated mice using the phenol red secretion test

After drug administration, ICR mice were fasted for 24 h with access to water. Subsequently, 15 mL of phenol red (1 mg/mL) mixed with 1 g of heated flour to form a paste was administered intragastrically. After 30 min, the mice were euthanized by decapitation; the stomach was dissected, and both the pylorus and antrum were ligated. The stomach was removed, and phenol red was eluted using cold physiological saline. The residual phenol red was quantified using a spectrophotometer to assess gastric emptying function. Gastric retention rate = (Absorbance of gastric residual pigment/Mean absorbance of gastric residual pigment in the control group) × 100%, and gastric emptying rate = 1 – Gastric retention rate.

### 2.5 Hematoxylin and eosin (H&E) staining

Muscle specimens from the left and right hind limbs of each group of C57BL/6 mice were selected, paraffin-embedded, sectioned, and stained with hematoxylin–eosin. The processed specimens were observed under an optical microscope and a polarizing microscope to examine the histopathological changes of the tissues.

### 2.6 Detection by Western blotting

Total proteins were extracted from each group of cells and the hind limb muscles of C57BL/6 mice. The concentration of the protein samples was determined using a BCA protein quantification kit and adjusted to a uniform concentration in accordance with the manual. 5X loading buffer was added to the equivalent samples and subjected to heat denaturation at 95°C for 5 min. The protein samples were electrophoretically separated on SDS-PAGE gel and then transferred to PVDF membranes. The membrane surfaces were blocked with 5% skimmed milk in TBST solution at room temperature for 1 h to minimize non-specific binding. Incubation was carried out overnight at 4°C with primary antibody (against AKT, p-AKT, pFoxo3a, MAFbx and MuRF1) diluent. The following day, the membranes were washed three times with TBST at room temperature for 10 min each time. Incubation with the corresponding horseradish peroxidase (HRP)-labeled secondary antibody was performed for 1 h at room temperature. The membranes were washed again three times with TBST for 10 min each time. Development was carried out using a chemiluminescent substrate (ECL). Data analysis: the protein bands were captured in the imaging using ChemiDoc MP Imaging System and the gray values of the protein bands were measured using Gel-Pro32 analysis software. The ratio of the gray values of the target proteins (AKT, p-AKT, pFoxo3a, MAFbx, MuRF1) to the internal reference (GAPDH) was calculated as the relative quantity of protein expression levels.

### 2.7 Extraction of primary gastric smooth muscle cells from constipated mice

In each group of ICR mice, after disinfecting the abdomen with 75% alcohol, the abdominal cavity was exposed. The stomach was cut off and immediately placed in ice-cold PBS containing double antibiotics. In a superclean bench, food residues and the inner membrane were carefully scraped off, leaving the muscular layer, which was rinsed 4–5 times with ice-cold PBS. After being cut into small pieces of approximately 1 mm x 1 mm with scissors, they were placed in a small vial pre-filled with approximately 3–4 mL of 1 g/L collagenase. The vial cap was tightened and placed in a 37°C water bath with stirring for digestion for 60 min, and the digestion fluid was collected every 20 min. The samples were centrifuged at 1,000 rpm to discard the supernatant, washed once with DMEM/F12 culture medium containing 10% FBS, and centrifuged at 1,000 rpm to discard the supernatant. Then, a cell suspension was prepared using DMEM/F12 culture medium (containing 10% FBS) and seeded into culture flasks, which were placed in a 37°C, 5% CO_2_ incubator for incubation. After adhering for 60–90 min, they were transferred to new flasks and the cell density was adjusted to 1 × 109/mL and placed in a CO_2_ incubator for static culture.

### 2.8 Detection of intracellular Ca^2+^ concentration in gastric smooth muscle cells of constipated mice

Fluo-3 AM was diluted with PBS at a ratio of 1:1000 to a final concentration of 1 μM. After the cell culture medium was removed, an appropriate volume of the diluted Fluo-3 AM was added, with the volume sufficient to fully cover the cells. Incubation was carried out in a 37°C cell culture incubator for 30 min. The cells were washed three times with PBS to completely remove the Fluo-3 AM that did not enter the cells. Fixation was performed with 4% formaldehyde for 10 min. An appropriate amount of DAPI staining solution was added, and staining was conducted for 5 min. The staining solution was discarded, and the cells were rinsed three times with PBS. The intracellular Ca^2+^ content of gastric smooth muscle cells was observed using laser confocal microscopy (Ex = 488 nm; Em = 530 nm).

### 2.9 Detection of ATP content in gastric smooth muscle cells of constipated mice

The gastric smooth muscle cells were washed with PBS, and the lysis buffer containing protease inhibitors was added and placed on ice for 10–15 min. The protein concentration in the lysate was determined by the BCA or Bradford method. Centrifugation was performed at 12,000 × *g* for 10 min, and the supernatant was collected for ATP determination. The ATP standard and enzymatic reaction mixture (such as luciferase and luminogenic substrate) were used for the reaction at room temperature or 37°C. The luminescence intensity was measured using a reader, and the ATP concentration in the cells was calculated. The ATP content was normalized to the protein concentration, and statistical analysis was conducted to compare different experimental groups.

### 2.10 Statistical analyses

Data were analyzed by GraphPad Prism 10.0 software. Multiple comparisons were performed with one-way analysis of variance (ANOVA) tests. The results were from at least triple independent experiments. All data were presented as mean ± standard deviation (SD) and *p* < 0.05 was considered statistically significant.

## 3 Results

### 3.1 Determination of ghrelin in mice

#### 3.1.1 Determination of ghrelin in mice with disuse muscular atrophy

The variations of ghrelin secretion levels in the serum of C57BL/6 mice in each group were examined. Compared with the control group, the ghrelin concentration in the model group declined, and the difference was statistically significant (*p* < 0.05). In contrast to the model group, the ghrelin concentration in the positive drug group rose, and the difference was statistically significant (*p* < 0.05). This suggests that the ghrelin level in the positive drug group truly elevated, and simultaneously validates that Shouhui Tongbian Capsules can cause an increase in ghrelin concentration, the results are shown in Figure 1.

#### 3.1.2 Determination of ghrelin content in constipated mice

The variations in ghrelin secretion levels in the serum of each group of ICR mice were as follows: Compared with the normal control group, the ghrelin level in the serum of the model group was lower than that of the normal control group (*p* < 0.05). Compared with the model group, the ghrelin contents in the serum of intervention group 1 and intervention group 2 were both higher than that of the constipation group (*p* < 0.05), the results are shown in Figure 2. Regarding the changes in ghrelin secretion in the gastric tissue: Compared with the normal control group, the ghrelin level in the gastric tissue of the model group was lower than that of the normal control group (*p* < 0.05). Compared with the model group, the ghrelin contents in the gastric tissue of intervention group 1 and intervention group 2 were both higher than that of the model group (*p* < 0.05), the results are shown in Figure 3.

### 3.2 Changes in general indicators of mice

#### 3.2.1 The effect of ghrelin on body weight changes and wet weight of hind limb muscles in different groups of mice with disuse muscle atrophy

**Figure 5:**
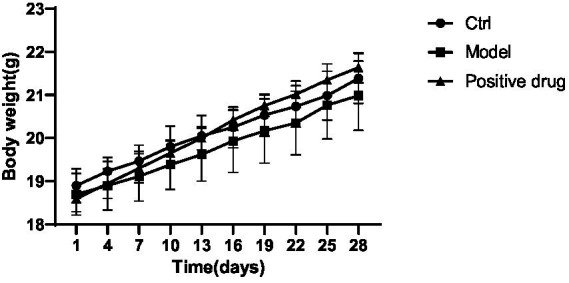
Body weight trends of mice in each group during the modeling period.

The body weight of the C57BL/6 mouse model group decreased in comparison with the blank control group, while the body weight of the positive drug group rebounded in contrast to the model group, the results are shown in Figure 5. The wet weight of hind limb muscles in the model group was conspicuously reduced compared to the blank control group. The differences in the wet weight of the left and right hind limb muscles were statistically significant (*p* < 0.01, *p* < 0.01). The wet weight of hind limb muscles in the positive drug group improved in relation to the model group. The difference in the wet weight of the left hind limb muscle was not statistically significant (*p* > 0.05), while the difference in the wet weight of the right hind limb muscle was statistically significant (*p* < 0.01), as presented in Table 1.

**Table 1:** Hindlimb wet weight in each group of disuse muscle atrophy mice (*x̄* ± *s*, *n* = 3).

Group	Wet weight of left hind limb muscle	Wet weight of right hind limb muscle
Blank control group	0.162 ± 0.018	0.183 ± 0.008
Model group	0.130 ± 0.017^***^	0.128 ± 0.011^*^
Positive drug group	0.150 ± 0.006^####^	0.157 ± 0.019^##^

^*^P < 0.05, ^***^p < 0.0001, compared with the blank control group. ^##^*p* < 0.01, ^####^*p* < 0.0001, compared with the model group.

#### 3.2.2 The effects of ghrelin on physiological indicators and food intake in constipated mice

The body indicators and food intake of each group of ICR mice during the modeling period and the drug intervention period were analyzed. As presented in Table 2, within the 0–21 days of the experiment, the body weights of each group of ICR mice fluctuated within the normal range, and there was no significant difference among the groups (*p* > 0.05). On the 21st day, the body weight of the mice in the model group was lower than that of the mice in the normal control group, and the difference was significant (*p* < 0.05); the body weight of the mice in the drug intervention groups was higher than that of the mice in the model group, and the difference was significant (*p* < 0.05). The defecation frequency, fecal particle count, wet weight of feces, and water content of feces of the mice in the model group were significantly lower than those of the mice in the normal control group, and all the differences were significant (*p* < 0.05). The dry weight of feces of the mice in the model group had no statistical difference from that of the mice in the normal control group (*p* > 0.05), indicating that the modeling of constipated mice in the experiment was successful and had a scientific basis. Meanwhile, the defecation frequency of the mice in the drug intervention groups was higher than that of the mice in the model group, and the difference was significant (*p* < 0.05); the fecal particle count of the mice in the drug intervention groups was higher than that of the mice in the model group, and the difference was significant (*p* < 0.05); the wet weight of feces of the mice in the drug intervention groups was higher than that of the mice in the model group, and the difference was significant (*p* < 0.05); the water content of feces of the mice in the drug intervention groups was higher than that of the mice in the model group (*p* < 0.05); while the dry weight of feces of the mice in the drug intervention groups had no statistical difference from that of the mice in the model group (*p* > 0.05). Regarding food intake, on the 21st day, the changes in food intake of each group of ICR mice were as follows. During the 0–1, 1–2, 2–4, and 4–24 h, the food intake of the mice in the model group was lower than that of the mice in the normal group (*p* < 0.05), while the food intake of the mice in the drug intervention groups was higher than that of the mice in the model group (*p* < 0.05).

**Table 2:** Changes in the general status in each group of constipated mice (*x* ± *s*, *n* = 6).

Item	Blank control (*n* = 6)	Model (*n* = 6)	Intervention1 (*n* = 6)	Intervention2 (*n* = 6)	*F*-value	*p*-value
Weight						
0 day	19.21 ± 0.10	19.08 ± 0.11	19.05 ± 0.15	19.25 ± 0.24	0.367	0.777
7 days	20.05 ± 0.166	20.01 ± 0.20	19.70 ± 0.12	19.95 ± 0.25	0.672	0.579
14 days	21.35 ± 0.18	20.43 ± 0.19	20.95 ± 0.199	21.45 ± 0.29	4.356	0.016
21 days	22.68 ± 0.20	20.93 ± 0.18	22.52 ± 0.265^#^	21.98 ± 0.177^#^	13.064	0.001
Frequency of defecation	19.50 ± 3.83	9.17 ± 2.13	15.33 ± 2.58^#^	16.33 ± 2.33^#^	14.30	0.001
Number of fecal particles	57.33 ± 3.35	30.83 ± 2.78	48.17 ± 2.97^#^	53.83 ± 3.45^#^	13.91	0.001
Wet weight of feces (g)	5.38 ± 0.29	2.87 ± 0.24	4.02 ± 0.24^#^	4.33 ± 0.188^#^	17.57	0.001
Dry weight of feces (g)	1.96 ± 0.80	1.85 ± 0.15	1.98 ± 0.11	1.88 ± 0.50	0.360	0.783
Water content of feces	63.29 ± 1.80	35.52 ± 1.46	50.30 ± 1.46^#^	56.36 ± 1.35^#^	59.74	0.001
Food intake on the 21st day						
0–1 h	0.94 ± 0.08	0.45 ± 0.51	0.695 ± 0.60^#^	0.69 ± 0.04^#^	13.90	0.001
1–2 h	1.19 ± 0.11	0.57 ± 0.42	0.95 ± 0.85^#^	1.05 ± 0.87^#^	13.06	0.001
2–4 h	1.85 ± 0.14	0.76 ± 0.65	1.21 ± 1.02^#^	1.47 ± 1.29^#^	22.82	0.001
4–24 h	7.01 ± 0.37	4.27 ± 0.30	6.51 ± 0.31^#^	6.70 ± 0.38^#^	13.35	0.001

^#^*P* < 0.05, compared with the model group.

#### 3.2.3 The effect of ghrelin on gastric emptying rate in constipated mice

**Figure 6:**
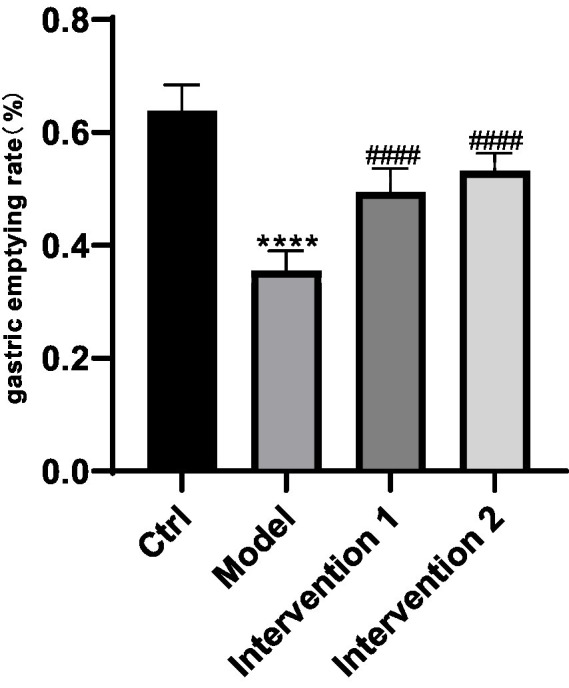
Comparison of gastric emptying rates among different mouse groups. ^****^p < 0.0001, compared with the blank control group; ^####^*p* < 0.0001, compared with the model group.

The variations in gastric emptying rates among each group of ICR mice: In contrast to the normal control group, the gastric emptying rate of the model group was markedly lower than that of the normal control group (*p* < 0.05). Compared with the model group, the gastric emptying rates of Intervention Group 1 and Intervention Group 2 were both higher than that of the model group (*p* < 0.05), the results are shown in Figure 6.

### 3.3 Pathological sections of skeletal muscles in each group of mice with disuse muscle atrophy

**Figure 7:**
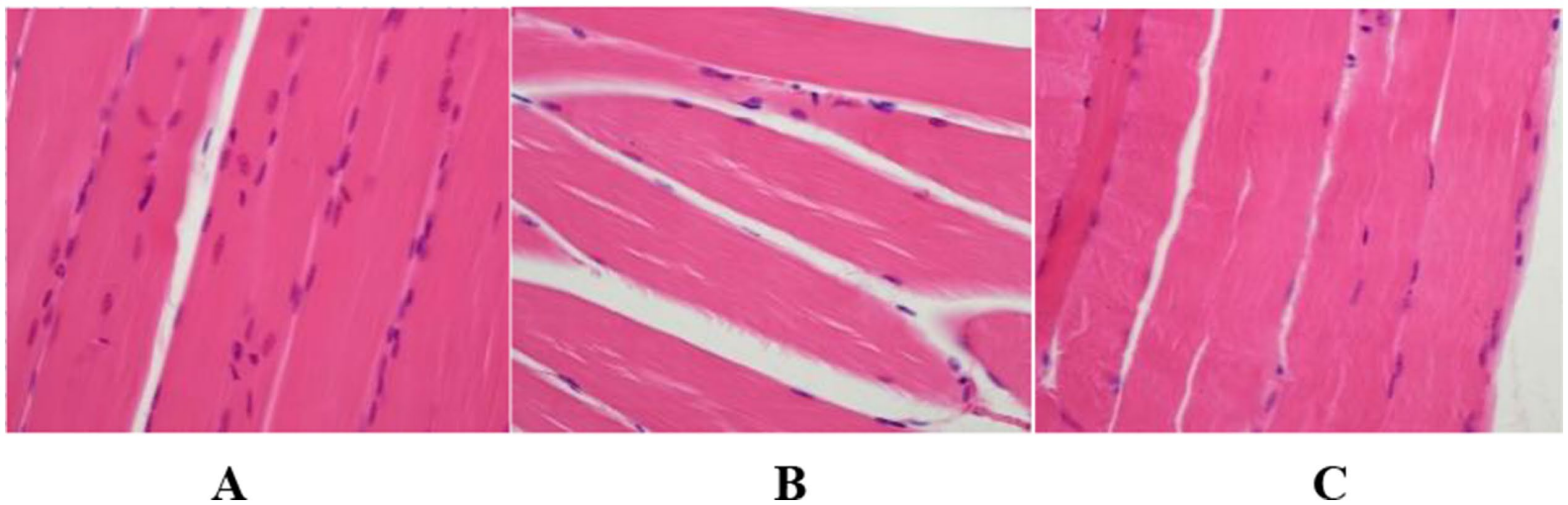
Histopathological findings of hind limb tissues in each group of mice (HE Staining, Scale Bar = 20 μm, 200×). **(A)** Blank control group; **(B)** model group; **(C)** positive drug group.

The hind limb tissues of each group of C57BL/6 mice were processed, and the results of HE staining revealed that in the model group, the striations of the skeletal muscle were ambiguous, the arrangement of muscle fibers was disordered with inconsistent directions, the muscle fiber bundles became thinner, and the intervals between the muscle bundles enlarged, indicating the successful establishment of the hind limb muscle atrophy model in the model group. In the positive drug group, the striations of the skeletal muscle were indistinct, the arrangement of muscle fibers was orderly, the directions of the majority of muscle fibers were consistent, the muscle fiber bundles became thinner, and the intervals between the muscle bundles decreased to some extent, suggesting that ghrelin has an ameliorative effect on muscle atrophy. The results are shown in Figure 7.

### 3.4 The effect of ghrelin on C2C12 myotubes *in vitro*

**Figure 8:**
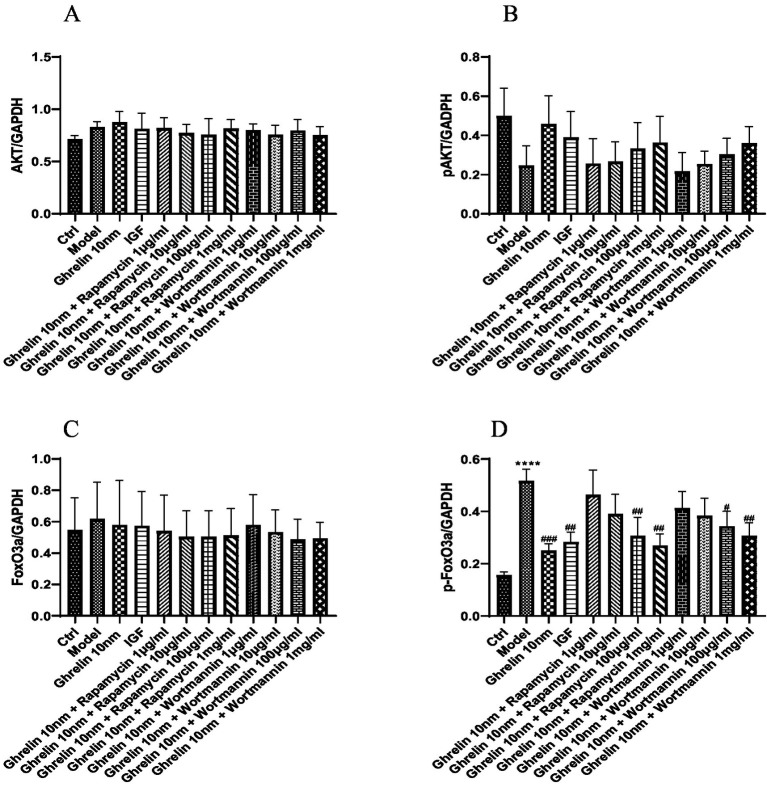
The expression of cellular proteins in each group was detected by Western blot analysis. **(A)** Expression tendency of AKT protein; **(B)** expression tendency of p-AKT protein; **(C)** the expression tendency of FoxO3a protein; **(D)** expression tendency of p-FoxO3a protein; ^****^p < 0.0001, compared with the blank control group; ^#^*p* < 0.05, ^##^*p* < 0.01, ^###^*p* < 0.001, compared with the model group.

The expression of AKT, p-AKT, Foxo3a, and p-Foxo3a, which are associated with the PI3K/AKT signaling pathway, was measured in 12 groups of cells using the Western blot technique. The results of the expression trend graphs for each protein are presented in Figure 8. The findings revealed that compared with the normal group, the expression of AKT protein increased, the expression of p-AKT protein decreased, the expression of Foxo3a protein increased, and the expression of p-Foxo3a protein increased in the model group (*p* < 0.0001). In contrast to the model group, the expression of p-AKT protein rose and the expression of p-Foxo3a protein declined in the ghrelin group (*p* < 0.001). Various concentrations of rapamycin were selected and added to the model group containing ghrelin. It was observed that in comparison with the model group, as the concentration of rapamycin increased, the expression of p-AKT protein gradually increased, but the difference was not significant (*p* > 0.05), while the expression of p-Foxo3a protein gradually decreased. Notably, there was a significant difference in the expression of p-Foxo3a protein at concentrations of 100 μg/mL and 1 mg/mL of rapamycin compared with the model group (*p* < 0.01). Different concentrations of wortmannin were incorporated into the model group containing ghrelin. Compared with the model group, except for the 1 μg/mL wortmannin group where the expression of p-AKT protein decreased, the expression of p-AKT protein increased in other concentration groups, but the difference was not significant (*p* > 0.05). In the analysis of p-Foxo3a protein, compared with the model group, as the concentration of wortmannin gradually increased, the expression of p-Foxo3a protein gradually decreased. Specifically, there was a significant difference in the expression of p-Foxo3a protein at a concentration of 100 μg/mL of wortmannin (*p* < 0.05).

### 3.5 The effect of ghrelin on the expression of atrophy-related proteins MAFbx and MuRF1

**Figure 9:**
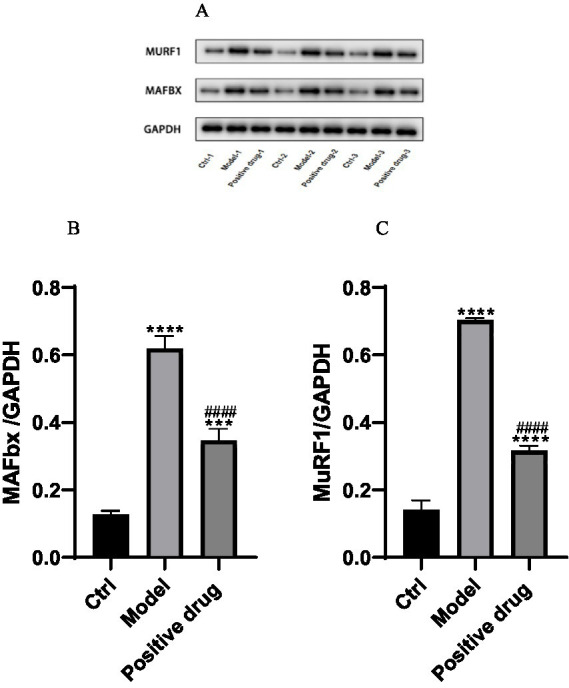
Expressions of MAFbx and MuFR1 in each group. ^***^p < 0.001, ^****^p < 0.0001, compared with the blank control group; ^####^*p* < 0.0001, compared with the model group.

The expression of muscle atrophy-related proteins in each group of C57BL/6 mice was examined. It was observed that the expression of muscle atrophy-related proteins MAFbx and MuFR1 in the model group was elevated compared to the blank group, and the differences were statistically significant (*p* < 0.01). After the intervention with positive drugs, the expression of MAFbx and MuFR1 was downregulated, and the differences were statistically significant (*p* < 0.01). That is, the intervention drugs were capable of increasing ghrelin and thereby inhibiting the expression of MAFbx and MuFR1. It was thus hypothesized that the positive drugs could ameliorate disuse muscle atrophy, as presented in Table 3. Subsequently, the expression levels of muscle atrophy-related proteins MAFbx and MuFR1 were analyzed and detected using the Western blotting method, shown in Figure 9A. The protein quantities were subjected to t-test histogram analysis, and a *p* < 0.05 was considered statistically significant, as shown in Figures 9B,C. The expression quantity of MAFbx protein was the highest in the model group, followed by that in the positive drug group; the expression quantity of MAFbx protein was the lowest in the normal control group. It could be seen that ghrelin in the positive drug group could suppress the expression of MAFbx protein. The expression quantity of MuFR1 protein was the highest in the model group, followed by that in the positive drug group; the expression quantity of MuFR1 protein was the lowest in the normal control group. It could be observed that ghrelin in the positive drug group was able to inhibit the expression of MuFR1 protein.

**Table 3:** Expression levels of MAFbx and MuRF1 in each group of disuse atrophy mice (*x̄* ± *s*, *n* = 3).

Group	Blank control	Model	Positive drug
MAFbx/GAPDH	0.13 ± 0.01	0.62 ± 0.04^****^	0.35 ± 0.04^####^
MuFR1/GAPDH	0.14 ± 0.03	0.70 ± 0.01^****^	0.32 ± 0.02^####^

****p < 0.0001, compared with the blank control group. ^####^*p*< 0.0001, compared with the model group.

### 3.6 The effect of ghrelin on intracellular Ca^2+^ content in constipated mice cells

**Figure 10:**
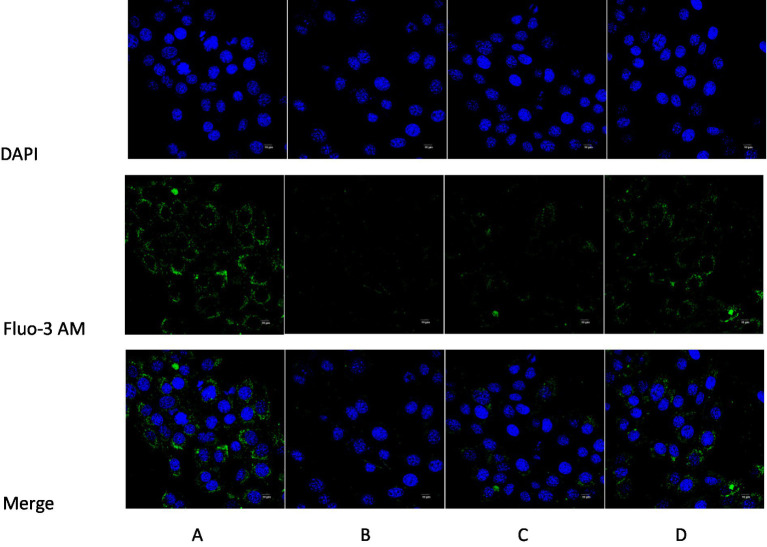
Comparison of intracellular Ca^2+^ content detected by Fluo-3 AM fluorescence probe in each group of mice. **(A)** Blank control group; **(B)** Model group; **(C)** Intervention 1 group; **(D)** Intervention 2 group.

The intracellular Ca^2+^ content of each group of ICR mice was determined using the Fluo-3 AM fluorescent probe. The blue fluorescence represented the cell nuclei stained by DAPI, the green fluorescence indicated Ca^2+^ stained by Fluo-3 AM, and the cyan fluorescence signified the fusion of the cell nuclei stained by DAPI and Ca^2+^ stained by Fluo-3 AM. In comparison with the normal control group, the intensity of the blue fluorescence in the model group was relatively lower. In contrast to the model group, the relative intensity of the blue fluorescence in intervention group 1 and intervention group 2 was stronger. Compared to the normal control group, the intensity of the green fluorescence in the model group was weaker. In opposition to the model group, the relative intensity of the green fluorescence in intervention group 1 and intervention group 2 was stronger. Compared with the normal control group, the intensity of the cyan fluorescence in the model group was lower. In contrast to the model group, the relative intensity of the cyan fluorescence in intervention group 1 and intervention group 2 was stronger; the results are shown in Figure 10.

### 3.7 The effect of ghrelin on ATP content in gastric smooth muscle cells of constipated mice

**Figure 11:**
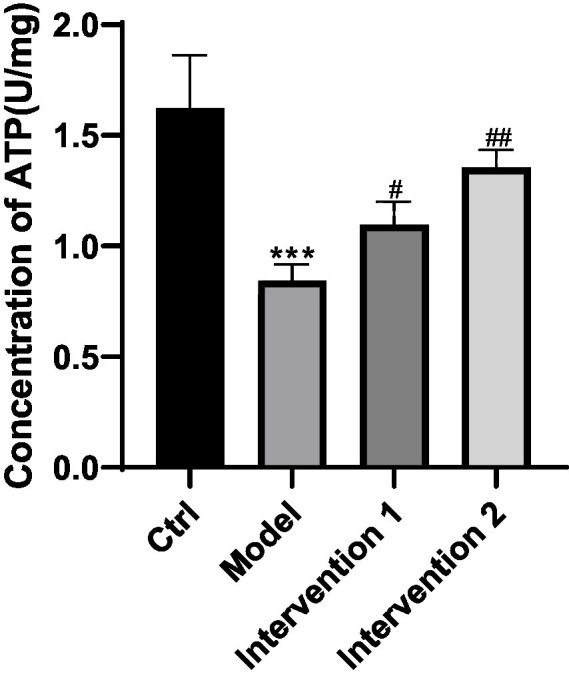
Comparison of ATP content in each group of mice4. ^***^p < 0.001, compared with the blank control group; ^#^*p* < 0.05, ^##^*p* < 0.01, compared with the model group.

The comparison of ATP content in gastric smooth muscle cells of ICR mice among each group indicated that the model group was lower than the normal control group (*p* < 0.05). In contrast to the model group, intervention group 1 and intervention group 2 were higher than the model group (*p* < 0.05), the results are shown in Figure 11.

## 4 Discussion

Constipation is a gastrointestinal motility disorder involving multiple factors, whose occurrence is closely associated with primary or secondary gastrointestinal dysmotility, imbalances of the intestinal microbiota, psychological factors, as well as adverse effects from diseases or medications. Skeletal muscle plays a crucial role in the body, and studies regarding its function and mechanisms are of significant importance for further exploration of digestive system diseases, including functional constipation. In recent years, increasing evidence has demonstrated a close association between skeletal muscle motor function and intestinal health (14, 15). Disuse muscle atrophy refers to the decrease in skeletal muscle mass and strength due to prolonged inactivity, such as extended bed rest, limb immobilization, or reduced physical activity. Especially in the elderly, studies have revealed a strong correlation between sarcopenia and disuse muscle atrophy, with both sharing highly consistent molecular mechanisms, each relying on the PI3K/Akt/mTOR signaling pathway as a core regulatory mechanism (16, 17). Sarcopenia significantly increases the risk of constipation, as it affects the entire skeletal musculature, leading to decreased strength and coordination of the pelvic floor muscles and reduced lumbar proprioception, thereby exacerbating defecatory disorders (18). In previous experiments, intravenous injection of ghrelin and normal saline in 12 healthy male animals demonstrated that ghrelin exerts a certain effect on gastric motility (19). Additionally, studies by Díaz-Lezama et al. (20) have shown that ghrelin can enhance Ca^2+^ channel expression, thereby increasing Ca^2+^ concentration, ultimately inducing smooth muscle contraction and promoting gastric emptying. Therefore, this study explores the mechanisms of ghrelin’s action on skeletal muscle and gastric smooth muscle, aiming to clarify its effects on improving skeletal muscle dysfunction and promoting gastrointestinal motility in mice.

Firstly, through *in vitro* experiments on disuse muscle atrophy, we found that ghrelin could activate the PI3K-Akt-mTORC1 pathway, promote downregulation of the muscle atrophy-related protein pFoxo3a, and induce an increase in pAkt protein levels, thereby playing a positive role in ameliorating disuse muscle atrophy. In a mouse model of disuse skeletal muscle atrophy established by tail-suspension, model mice showed significantly decreased body weight and hindlimb muscle wet weight, together with significantly increased expression of muscle atrophy-related proteins compared to controls. As a well-established method, the tail-suspension model in this experiment showed significantly decreased ghrelin levels, which may be related to oxidative stress resulting from disuse atrophy (21). Under oxidative stress, decreased ghrelin levels may reflect the organism’s response to balance energy demands (22). Meanwhile, after intervention with Shouhui Tongbian Capsule, ghrelin levels in mice markedly increased compared to the model group, but remained below those in the normal control group. The possible reason is that, while Shouhui Tongbian Capsule can partially restore ghrelin expression, complete recovery in a diseased state is still influenced by oxidative stress and energy metabolism regulation. Further experimental results showed a significant reduction in muscle protein synthesis capacity in the model group. After intervention with the positive control drug Shouhui Tongbian Capsule, ghrelin levels increased and the phenotype of muscle atrophy was significantly improved. Specifically, compared to the model group, mice in the positive drug group experienced less weight loss, increased hindlimb muscle wet weight, downregulation of muscle atrophy-related proteins MAFbx and MuRF1, and improvements in pathological features.

Secondly, this study also explored the role of ghrelin in a loperamide-induced constipation mouse model and evaluated the effects of different interventions on gastrointestinal function. Comparison of ghrelin content in gastric tissue and serum among groups revealed that ghrelin levels in both the model and intervention groups 1 and 2 were significantly lower than in the normal control group. Although the two intervention groups showed increased ghrelin levels compared to the model group, recovery to normal levels was not observed. Possible mechanisms include: decreased gastrointestinal peristalsis and delayed gastric emptying in constipation, resulting in reduced mechanical stimulation of the gastric mucosa, thereby affecting ghrelin secretion; additionally, gastrointestinal inflammation and neuroendocrine dysregulation may inhibit ghrelin expression. Meanwhile, loperamide, as an opioid receptor agonist, further decreases ghrelin levels by suppressing gastrointestinal neural reflexes and hormone secretion. Therefore, although intervention drugs can increase ghrelin levels to some extent, the restoration of ghrelin secretion under pathological or pharmacologically suppressed conditions remains limited. This indirectly reflects the important role of ghrelin in the occurrence and development of constipation, suggesting it may serve as a pathophysiological biomarker and potential intervention target for constipation. Experimental results further showed that, compared to the normal control group, model mice exhibited significant reductions in body weight, defecation frequency, number of fecal pellets, fecal wet weight and water content, but there was no significant change in fecal dry weight, indicating successful model establishment and reduced food intake. Further analysis revealed that gastric emptying rate, intracellular Ca^2+^ and ATP content in gastric smooth muscle cells in the model group were also significantly lower than those in the normal group, demonstrating that gastrointestinal motility, energy metabolism, and calcium signaling pathways are all impaired, which may further affect smooth muscle contraction and overall gastrointestinal function. Following intervention, both intervention group 1 and group 2 mice showed significant increases in body weight and food intake, as well as higher defecation frequency, number of fecal pellets, wet weight, and water content of feces compared to the model group, with group 2 exhibiting a greater degree of improvement. It is worth noting that there was no difference in fecal dry weight between the intervention groups and the model group, suggesting that the interventions primarily improved water content and gastrointestinal motility. Additionally, gastric emptying rate was significantly elevated with intervention, particularly in group 2. On the molecular level, both intervention groups significantly increased intracellular Ca^2+^ and ATP content, with group 2 showing more pronounced improvement. Restoration of Ca^2+^ and ATP facilitates enhanced gastric smooth muscle contractility, thereby improving gastrointestinal motility and defecation function. Therefore, Shouhui Tongbian Capsule may effectively improve gastrointestinal function and defecation capability in constipated mice via multiple mechanisms, including elevating ghrelin levels, regulating intracellularCa^2+^ dynamics, promoting ATP synthesis, and enhancing smooth muscle contraction. Its overall efficacy surpassed that of domperidone.

In summary, our study demonstrates that pharmacological increase of ghrelin not only ameliorated skeletal muscle dysfunction induced by disuse atrophy, but also enhanced gastric smooth muscle contractility and improved gastrointestinal motility, thereby effectively alleviating constipation. Considering the highly consistent molecular mechanisms between disuse muscle atrophy and sarcopenia, this suggests that ghrelin may also have potential therapeutic effects on constipation resulting from sarcopenia-induced muscle dysfunction. Our study highlights the important role of ghrelin in improving skeletal muscle disuse atrophy and gastrointestinal motility in animal models of constipation, indicating that ghrelin upregulation may be an effective strategy for intervening in skeletal muscle dysfunction and constipation. However, it should be emphasized that constipation is a complex syndrome involving multiple factors and pathways. Its pathogenesis is not only regulated by skeletal muscle function and ghrelin, but also by 5-HT signaling, the gut microbiota, dopamine D2 receptors, and various other factors. Previous studies have reported that Shouhui Tongbian Capsule can improve intestinal function by modulating the 5-HT system and the gut microbiota (23), while domperidone mainly acts on gastrointestinal motility by antagonizing dopamine D2 receptors (24). Given the close relationship between ghrelin and gastric emptying, the increase in ghrelin observed in this study may be both a consequence of enhanced gastrointestinal motility and a promoter of further intestinal peristalsis, and the causal sequence warrants further investigation. Therefore, the mechanisms underlying the effect of Shouhui Tongbian Capsule on constipation are likely the result of multiple pathways acting synergistically and interactively, with ghrelin being just one important factor rather than the sole driver. Future research should explore the causal relationships among individual signaling pathways and molecules through experiments such as specific pathway inhibition, microbiota transplantation, and temporal tracking. A limitation of this study is that we were unable to fully distinguish between the independent and synergistic effects of different pathways; subsequent investigations will address this issue.

## 5 Conclusion

Through systematic experiments and data analysis, this study is the first to elucidate the significant role of ghrelin in the gastric smooth muscle of disuse muscle atrophy mice with concomitant constipation. The results demonstrated that ghrelin upregulates the PI3K/AKT signaling pathway, significantly reduces the expression of atrophy-related proteins MAFbx, MURF-1, and FOXO3a, increases ATP content and Ca^2+^ concentration, and enhances the contractile capacity of smooth muscle cells, thereby improving overall muscle cell function. Furthermore, pharmacological elevation of ghrelin levels not only alleviated skeletal muscle dysfunction induced by disuse atrophy, but also enhanced the contractility of gastric smooth muscle, improved gastrointestinal motility, and effectively relieved constipation.

Given the overlap in molecular mechanisms between disuse muscle atrophy and age-related sarcopenia, the findings of this study suggest that ghrelin may also exert potential therapeutic effects on constipation associated with sarcopenia-induced muscle dysfunction. Regulation of ghrelin levels may provide a novel strategy for intervention in muscle dysfunction, gastrointestinal motility disorders, and associated constipation. It should be noted that the pathogenesis of constipation is multifactorial and involves multiple pathways. Although ghrelin plays an important role, it is not the sole determinant. Thus, future studies are warranted to further investigate the interactions between ghrelin and other signaling pathways or molecular factors, thereby offering more comprehensive insights for the prevention and treatment of constipation and other gastrointestinal disorders. These findings not only present new perspectives for clinical therapy, but also align with the holistic medical tenet of preventive treatment.

## Data Availability

All the data generated and analyzed in this study are original experimental data and not derived from public databases. These data are not deposited in a public repository but are available from the corresponding author upon reasonable request.
